# Malaria incidence and efficacy of intermittent preventive treatment in infants (IPTi)

**DOI:** 10.1186/1475-2875-6-163

**Published:** 2007-12-09

**Authors:** Robin Kobbe, Samuel Adjei, Christina Kreuzberg, Benno Kreuels, Benedicta Thompson, Peter A Thompson, Florian Marks, Wibke Busch, Meral Tosun, Nadine Schreiber, Ernest Opoku, Ohene Adjei, Christian G Meyer, Juergen May

**Affiliations:** 1 Infectious Disease Epidemiology Group, Bernhard Nocht Institute for Tropical Medicine, Bernhard-Nocht-Straße 74, D-20359 Hamburg, Germany; 2 Ministry of Health/Ghana Health Service District Health Directorate, Agona, Ashanti Region, Ghana; 3 Kumasi Centre for Collaborative Research in Tropical Medicine, Kumasi, Ghana; 4 International Vaccine Institute, Seoul, South Korea; 5 Dept. Molecular Medicine, Bernhard Nocht Institute for Tropical Medicine, Hamburg, Germany

## Abstract

**Background:**

Intermittent preventive antimalarial treatment in infants (IPTi) is currently evaluated as a malaria control strategy. Among the factors influencing the extent of protection that is provided by IPTi are the transmission intensity, seasonality, drug resistance patterns, and the schedule of IPTi administrations. The aim of this study was to determine how far the protective efficacy of IPTi depends on spatio-temporal variations of the prevailing incidence of malaria.

**Methods:**

One thousand seventy infants were enrolled in a registered controlled trial on the efficacy of IPTi with sulphadoxine-pyrimethamine (SP) in the Ashanti Region, Ghana, West Africa (ClinicalTrial.gov: NCT00206739). Stratification for the village of residence and the month of birth of study participants demonstrated that the malaria incidence was dependent on spatial (range of incidence rates in different villages 0.6–2.0 episodes/year) and temporal (range of incidence rates in children of different birth months 0.8–1.2 episodes/year) factors. The range of spatio-temporal variation allowed ecological analyses of the correlation between malaria incidence rates, anti-*Plasmodium falciparum *lysate IgG antibody levels and protective efficacies provided by IPTi.

**Results:**

Protective efficacy of the first SP administration was positively correlated with malaria incidences in children living in a distinct village or born in a distinct month (R^2 ^0.48, p < 0.04 and R^2 ^0.63, p < 0.003, respectively). Corresponding trends were seen after the second and third study drug administration. Accordingly, IgG levels against parasite lysate increased with malaria incidence. This correlation was stronger in children who received IPTi, indicating an effect modification of the intervention.

**Conclusion:**

The spatial and temporal variations of malaria incidences in a geographically and meteorologically homogeneous study area exemplify the need for close monitoring of local incidence rates in all types of intervention studies. The increase of the protective efficacy of IPTi with malaria incidences may be relevant for IPTi implementation strategies and, possibly, for other malaria control measures.

## Background

Effective antimalarial control strategies for young children in Africa must urgently be improved, as this group is at highest risk of morbidity and mortality due to *Plasmodium falciparum *malaria [1,2]. In most parts of sub-Saharan Africa, individual control measures concentrate on the reduction of parasite transmission through insecticide-treated bed nets and treatment of acute malaria episodes [3], the latter often being inappropriate due to increasing parasite drug resistance [4,5]. In addition to established control measures [6], novel approaches comprise pre-erythrocytic stage vaccines and the concept of "intermittent preventive treatment in infants" (IPTi) [7,8].

IPTi essentially exerts therapeutic and prophylactic effects of antimalarial drugs administered at intervals short enough to prevent disease, but long enough to allow for the development of protective immunity [9]. However, the exact mechanisms of protection are still not clear. It has been assumed that an optimal drug application schedule depends on the half-life of the antimalarial drugs used, the extent of current parasite drug-resistance, the transmission intensity, and the incidence of malaria episodes [10].

The IPTi application schedule that was originally evaluated in Tanzania was based on weight-adapted single-dose sulphadoxine-pyrimethamine (SP) and was pragmatically linked to the expanded programme on immunization (EPI) recommended by WHO, with vaccinations at months 2, 3 and 9 [8]. In that study, IPTi was 59% effective in reducing malaria episodes in an area with an incidence rate (IR) of 0.43 per person-year. In subsequent trials performed in Northern Ghana and Mozambique, protective efficacies were 24.8% (IR 1.02) and 22.2% (IR 0.55), respectively [11,12]. So far, studies modelling the impact of the malaria incidence on the protection that is provided by IPTi are not available.

The linear regression model presented here makes use of data of a recent controlled trial on SP-based IPTi performed in Ghana. The overall protective efficacy in this trial was 20.3% (IR 1.20) and decreased with the age of study participants [13]. The study presented here aims to determine the influence of spatio-temporally different malaria incidences on the efficacy of IPTi with SP in an ecological analysis.

## Methods

### Study area

The trial was conducted from January 2003 to September 2005 in the Ashanti Region, Ghana, West Africa, in nine neighboring villages of the rural Afigya Sekyere district. The district occupies an area of 714 km^2 ^in the forest belt. Geographically, the study area is homogeneous [14]. All distances between villages are < 15 km with altitude levels between 300 and 500 metres above the sea level. Temperature varies between 20.4°C and 33.5°C while monthly rainfall ranges from 15 mm (January) to 214 mm (June) with similar fluctuation in all villages. The area is holoendemic for *P. falciparum *malaria with perennial transmission and seasonal peaks (high transmission from May to October) [15]. Malaria vectors are mosquitoes of the *Anopheles gambiae *complex and *Anopheles funestus*. The peak entomological inoculation rate (EIR) of *P. falciparum *is > 100 infectious bites per individual in October. While chloroquine was the first-line drug for treatment of uncomplicated malaria in Ghana at the beginnning of the study, the Ghana Health Services has recommended a three-day regimen of amodiaquine-artesunate since March 2003. *Plasmodium falciparum *resistance against chloroquine and SP in the study area is high [16].

### Study design

One-thousand seventy infants at the age of three months (tolerance of four weeks accepted) with permanent residence in one of the villages under survey were recruited throughout the year 2003 [13]. In average 89 infants (range 59–110) were enrolled monthly and randomized to receive either a single-dose of 250 mg sulphadoxine and 12.5 mg pyrimethamine or placebo (verum drug and placebo provided by Roche, Basel, Switzerland). IPTi-treatment doses were given at the age of 3, 9 and 15 months (IPTi-1, IPTi-2, IPTi-3, respectively). Monthly follow-up visits were made over a period of 21 months until the age of 24 months. A structured questionnaire and a physical examination were completed and documented on each consultation. Clinical malaria episodes were defined as events of fever (rectal body temperature ≥ 38.0°C or fever reported to have occurred during the preceding 48 h) together with asexual *P. falciparum *parasitaemia (> 500 parasites/μl).

Examination of blood films followed quality-controlled standardized procedures. Briefly, films where air-dried, Giemsa-stained and independently read twice by two laboratory technicians by light microscopy. Parasitaemia levels were determined by scoring the number of asexual stage parasites per 200 leukocytes. If less than 10 parasites per 200 leukocytes were counted, the parasitaemia level was related to 500 leukocytes. Parasite densities were converted to the number of parasites per μL of blood, assuming 8,000 leukocytes/μL. Blood films were read again in case of ambiguities of parasite positivity/negativity, parasitaemia levels (ratio > 3), or of the parasite species. The final parasitaemia used for further statistical analyses was the median parasitaemia of all readings that were rated positive.

IgG antibody levels against crude *P. falciparum *lysate were measured by enzyme linked immunosorbent assays (ELISA) in all participants who completed follow-up as described in detail previously [17]. The parasite lysate fraction used as antigen was prepared from cultures of infected erythrocytes isolated from children with severe malaria living in the same malaria endemic region and results were expressed in Relative Units (RU) of IgG antibody levels.

### Ethical considerations

Before enrolment of participants written or thumb-printed (in the presence of an unbiased witness) parental consent was obtained. All clinical investigations within the core study were conducted according to the principles expressed in the Declaration of Helsinki. The protocol was approved by the Committee of Human Research, Publications and Ethics, School of Medical Sciences, Kwame Nkrumah University of Science and Technology, Kumasi, Ghana. The trial was registered at Clinical Trial Registration System with the number NCT00206739.

### Statistical analysis

Data from structured questionnaires and forms were entered within five days after each visit into a database (4th Dimension, Paris, France). Data were cross-checked by a study physician before files were locked. All information on participants and their parents was strictly confidential. Copies of all data and the original source documents were retained. Analyses were performed with the STATA/SE software, version 9.2 (College Station, TX, USA).

Malaria incidence rates were calculated for children of the placebo group for the period of one year, beginning at the time of recruitment. Children were not rated at risk for malaria for 21 days after preceding malaria episodes or after antimalarial treatment. Protective efficacy was calculated for the period beginning at recruitment and ending three months after IPTi-3, or, for time stratification, for periods of six months after each SP administration. Protective efficacy against multiple malaria episodes was determined by Poisson regression and defined as one minus rate ratio.

Time-dependency of protective efficacy was assessed by tests on violation of proportional hazards assumptions which were graphically displayed in log-minus-log plots for each category of a nominal covariate versus log(analysis time) and formally tested by Schoenfeld residuals [18]. A multivariate Poisson regression model with time-dependent covariates defined by six-months periods after each IPTi dose was generated, and effect modification of protective efficacy with time strata was evaluated by log-likelihood tests.

Malaria incidence rates and protective efficacies were stratified for the month of birth and the village of residence of participants. Incidence rates were plotted against the protective efficacy or against IgG antibody levels of the same stratum and the unweighted regression coefficient was calculated.

## Results

### Basic data

The age distribution, sex ratio, number of parasitaemic infants at recruitment and the extent of bed net usage did not differ significantly between the treatment and the placebo group (Table 1).

**Table 1:** Characteristics of study participants

Characteristic	Sulphadoxine-pyrimethamine n = 535	Placebo n = 535	p†
Sex, no.			
Male	264 (49.3%)	272 (50.8%)	0.63
Female	271 (50.7%)	263 (49.2%)	
Age in weeks at recruitment, mean (SD)			
Age at IPTi-1*	12.3 (± 1.7)	12.3 (± 1.6)	0.85
Use of bednets, no.			
Yes	210 (39.3%)	201 (37.6%)	
No	261 (48.8%)	263 (49.2%)	0.75
Missing data	64 (12.0%)	71 (13.3%)	
*P. falciparum *parasitaemia at recruitment, no.			
Positive	72 (13.5%)	86 (16.1%)	
Negative	463 (86.5%)	448 (83.8%)	0.29
Missing data	0 (0.0%)	1 (0.2%)	

SD, standard deviation.

* Age at IPTi-1 is equal to age at time of recruitment.

† By Wilcoxon or χ^2 ^test, none of the parameters in both study arms was significantly different.

### Malaria incidence and date of birth

**Figure 1:**
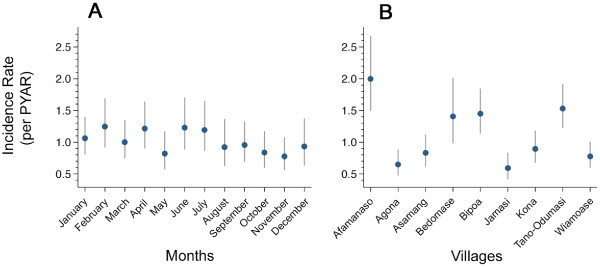
**Stratified malaria incidence rates**. Malaria incidence rates per PYAR in the placebo group assessed over the period of one year, beginning at the time of recruitment. A, PYAR stratified for the month of birth of the children; B, PYAR stratified for the village of residence of the children. PYAR, person year at risk.

During the first year of the follow-up period, incidence rates (IR) of malaria were influenced by the month of birth of children (Figure 1A). IR was highest among children born in February (IR 1.2 episodes/year, 95% CI 0.9–1.7) and tended to decrease in children born during the second half of the year. The lowest malaria IR was observed in children born in November (IR 0.8 episodes/year, 95% CI 0.6–1.1).

### Malaria incidence and village of residence

During the first year of follow-up visits, IRs were strongly dependent on the village of residence (Kruskall-Wallis test p < 0.001) (Figure 1B). The highest IR of 2.0 episodes/year (95% CI 1.5–2.7) was observed in the village Afamanaso, and the lowest IR of 0.6 episodes/year (95% CI 0.4–0.8) was found in Jamasi.

### IPTi schedule and protective efficacy of IPTi

Tests on the assessment of the proportional hazards assumption indicated that the protective efficacy of IPTi was dependent on the age of children at the time of IPTi application. First, vertical differences on log-minus-log survival plots for treatment and placebo tended to be higher in the first half of the observation period. Second, the plots of Kaplan-Meier survival curves of the observed data and the predicted Cox curves deviated considerably in the first year of the observation period in both groups. Third, Schoenfeld residuals provided evidence for violation of the proportional hazards assumption (rho 0.17, p = 0.007).

The evidence for violation of the proportional hazards assumption indicates that protective efficacies depend on the age of participating children at the time of of IPTi administration. Accordingly, analyses of the impact of IRs on protective efficacies were stratified for periods of six months after IPTi-1, IPTi-2 and IPTi-3 [13]. IPTi-1 administered at month 3 was more efficacious [23% protective efficacy (CI 6%–36%)] than IPTi-2 administered at nine months of age [17% (CI 1%–31%)] and IPTi-3 administered at 15 months of age [-5% (CI -25%–11%)].

### Malaria incidences and protective efficacies

**Figure 2:**
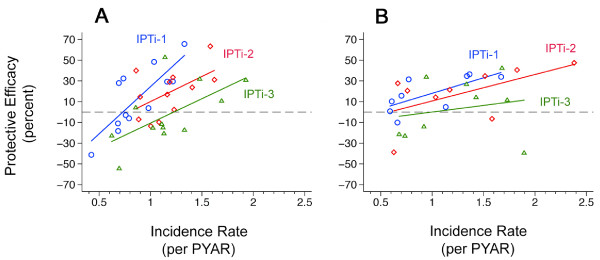
**Malaria incidence rates and protective efficacy**. Correlation between the protective efficacy of each IPTi application during six months after the drug administration (blue circles and line, after IPTi-1; red diamonds and line, after IPTi-2; green triangles and line, after IPTi-3) and malaria incidence rates per PYAR for the same time periods in the placebo arm. A, stratified by month of birth (IPTi-1, R^2 ^0.63, p < 0.003; IPTi-2, R^2 ^0.29, p = 0.07; IPTi-3, R^2 ^0.37, p < 0.04); B, stratified by village of residence (IPTi-1, R^2 ^0.48, p < 0.04; IPTi-2, R^2 ^0.33, p = 0.11; IPTi-3, R^2 ^0.04, p = 0.60). PYAR, person year at risk.

After stratification for the month of birth, the protective efficacies of IPTi-1 and IPTi-3 correlated significantly with the IRs (R^2 ^0.63, p < 0.003 and R^2 ^0.37, p < 0.04, respectively) (Figure 2A). A trend of a corresponding correlation was observed after IPTi-2 (R^2 ^0.29, p = 0.07). In accordance to the results obtained for the month of birth, protective efficacies correlated significantly with IRs after stratification for the villages of residence. This applied to the efficacy of IPTi-1 (R^2 ^0.48, p < 0.04) and, as a tendency, to that of IPTi-2 (R^2 ^0.33, p = 0.11) (Figure 2B).

### Malaria incidence and IgG antibody levels

**Figure 3:**
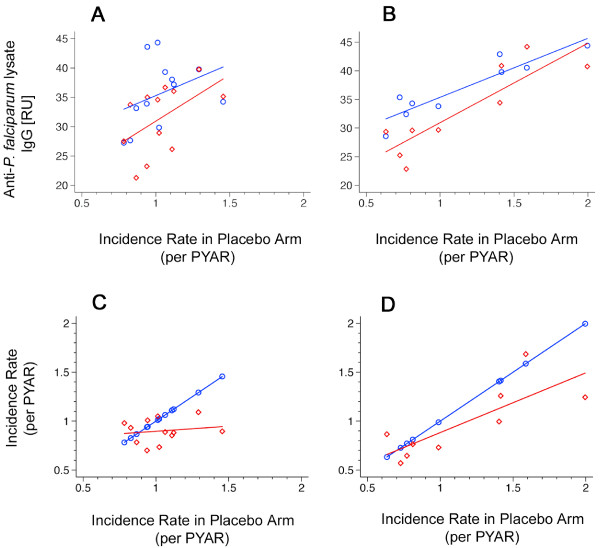
**Malaria incidence rates and anti-P. falciparum lysate IgG levels I**. Panel A and B, correlation between the mean of anti-*P. falciparum *lysate IgG levels and malaria incidence rates in the placebo arm. A, stratified by month of birth (red diamonds and lines [linear regression, R^2 ^0.54, p < 0.006], children from the SP arm; blue circles and lines [linear regression R^2 ^0.05, p = 0.475], children from the placebo arm. B, stratified by village of residence (red diamonds and lines [linear regression, R^2 ^0.90, p < 0.001], children from the SP arm; blue circles and lines [linear regression, R^2 ^0.89, p < 0.001], children from the placebo arm). Panel C and D, correlation between malaria incidence rates and incidence rates in the placebo arm. C, stratified by month of birth (red diamonds and lines [slope 0.07, p < 0.80], children from the SP arm; blue circles and lines, children from the placebo arm as comparison [slope 1]). D, stratified by village of residence (red diamonds and lines [slope 0.67, p < 0.007], children from the SP arm; blue circles and lines, children from the placebo arm as comparison [slope 1]). PYAR, person year at risk.

**Figure 4:**
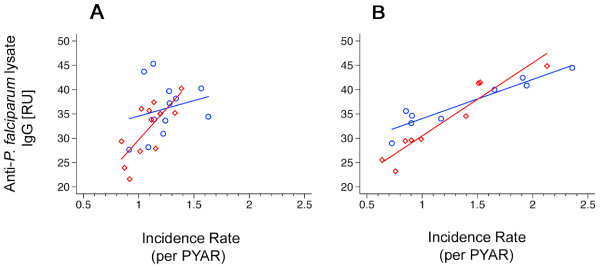
**Malaria incidence rates and anti-P. falciparum lysate IgG levels II**. Correlation between the mean of anti-*P. falciparum *lysate IgG levels and malaria incidence rates of the respective study arm in each village. A, stratified by month of birth (Wald test, p = 0.118). B, stratified by village of residence (Wald test, p < 0.001). Blue circles and lines (linear regression), children from placebo arm; red diamonds and lines (linear regression), children from SP arm. PYAR, person year at risk.

In children who received SP, mean IgG antibody responses against crude parasite lysate increased strongly with malaria IR in the same stratum (stratified by month of birth, R^2 ^0.54, p < 0.006; stratified by village, R^2 ^0.90, p < 0.001) (Figure 3A, B). In the placebo arm, this correlation was weaker than in children who received SP indicating an effect modification (stratified by month of birth, R^2 ^0.05, p = 0.475; stratified by village, R^2 ^0.89, p < 0.001). In contrast, the slope of malaria IR in the SP arm was lower (stratified by month of birth, slope 0.07, p < 0.80; stratified by village, slope 0.67, p < 0.007) than in the placebo group demonstrating the increase of protective efficacy with IR (Figure 3C, D). The Wald test for this interaction was significant for villages of residence (p < 0.001) and showed a similar trend when stratifying for the months of birth (p = 0.118) (Figure 4A, B).

## Discussion

The data from the ecological analysis show that the extent of protection provided by IPTi increased almost linearly with the malaria incidence. This applied to spatial and temporal stratifications. The strongest correlation between IPTi efficacy and malaria incidence was observed after IPTi-1, namely during the period of six months when treatment was most efficacious, but the correlation still held after IPTi-2 and, to a certain extent, after IPTi-3 as well.

Protective efficacy is a relative measure that is independent of incidences. Therefore, a higher malaria incidence *per se *cannot explain a higher protective efficacy. An appealing explanation for the observation that the protective efficacy of IPTi increased with the malaria incidence could be the additional influence of acquired immunity, which is also dependent on the incidence of malaria. Indeed, the level of anti-*P. falciparum *IgG, which may be considered a measure of exposure [19], increased in both treatment arms with the number of clinical malaria attacks. In addition, IgG levels were non-proportionally elevated in children who received SP in villages with higher malaria incidences. Although the crude anti-*P. falciparum *IgG level is only a weak proxy measure for acquired anti-malaria immunity, it reflects the cumulative parasitological challenge and, indirectly, the extent of semi-protective immunity [17,19-21].

According to the findings reported here, spatial differences of the *in vivo *efficacy of antimalarial treatment have previously been described from Uganda [22], where the efficacy of SP, in combination with other antimalarial compounds, also increased with transmission intensity, indicating a strong influence of immunity on treatment response.

Not consistent with the observation of a positive correlation between malaria incidences and IPTi-mediated protective efficacies is the fact that, in the aforementioned study from Tanzania, the relative protective efficacy was, with 59%, higher than that in our study, although the malaria incidence was, with 0.43 per person per year, lower [8]. This inconsistency may result from varying degrees of SP drug resistance, differences in the usage of insecticide-treated bed nets, and variations of the study protocols, such as other drug application schedules and case-finding strategies [23]. The absolute reduction of the number of malaria episodes was almost identical in the Tanzanian trial, but also in that from northern Ghana and in the study presented here (incidence rate reduction 0.26, 0.25 and 0.26 episodes per year, respectively) [8,11,13]. An exception was the trial from Mozambique, where the absolute episode reduction was, with an annual incidence rate reduction of 0.11 episodes, lower [12].

Although the geographical locations, altitudes and climatic conditions of villages were largely homogeneous throughout the study area, a number of parasite and host-dependent factors might account for the micro-epidemiological differences between neighbouring villages observed here [14]. High variation of malaria transmission intensities with regard to season and geography are known to occur across Africa [24]. In contrast, inter-village differences of malaria incidences have only rarely been studied [25], although mathematical modelling has identified environmental heterogeneity as a pivotal factor for the implementation of malaria control programmes and the development of immunity [26-28].

In this study, infants born in November were given drugs in February during the low-transmission season. The association of seasonal malaria incidences with protective efficacy of IPTi may explain why in areas of high seasonal transmission the administration of antimalarials was most effective at times of intense malaria transmission [29]. Supporting evidence comes from the IPTi study in northern Ghana where transmission is highly seasonal and children received four doses of SP at times of routine EPI vaccinations [11]. Although overall protection against clinical malaria was below 25% in that study, a subgroup of children who received first and second IPTi doses during the high transmission season were protected by more than 50%.

## Conclusion

The data presented here indicates that the efficacy of IPTi crucially depends on the differential spatial and temporal malaria incidence. This observation has implications on implementation strategies of IPTi and, most likely, also on other malaria control measures. More generally, the data show that even in an apparently homogeneous study  area, considerable variation may   exist, which may have an impact on the outcome of intervention studies. Cost-effectiveness analyses and mathematical models should consider the dependency of malaria incidences on efficacy and control interventions should be adapted to the wide range of transmission conditions [30-32]. Since efficient control measures will reduce malaria incidences, control measures must constantly be adapted to maintain their protective efficacy. The development and application of appropriate tools, such as satellite information and broad coverage of communication of relevant geographical, meteorological and micro-epidemiological data should be enforced in order to predict malaria dynamics and support the choice of appropriate control measures.

## Abbreviations

C – Celsius

CI – Confidence Interval

EIR – entomological inoculation rate

ELISA – linked immunosorbent assays

EPI – expanded program on immunization

IgG – immunglobuline G

IPTi – intermittent preventive treatment in infants

IPTi-1 – SP treatment given at the age of 3 months

IPTi-2 – SP treatment given at the age of 9 months

IPTi-3 – SP treatment given at the age of 15 months

IR – malaria incidence rate

km – kilometre

mg – milligram

mm – millimetre

μl – microlitre

RU – Relative Units

R^2 ^– correlation coefficient

SP – sulphadoxine-pyrimethamine

WHO – World Health Organization

## Competing interests

The author(s) declare that they have no competing interests.

## Authors' contributions

All authors participated in design, implementation, analysis or interpretation of the study. JM and CGM designed the study. JM and RK were involved in all phases of the study and have full access to all the data in the study. JM takes responsibility for the integrity of the data and the accuracy of the data analysis. OA was the Principle Investigator and supervised the study. RK, SA and CK were responsible for conduction of field studies and coordination of study procedures. Further acquisition and analysis of data was performed by WB, BK, NS and FM. Analysis of data and drafting of the manuscript was led by RK, WB and JM. CGM critically reviewed the manuscript and substantial input came from all investigators.

## References

[B1] Snow RW, Guerra CA, Noor AM, Myint HY, Hay SI (2005). The global distribution of clinical episodes of *Plasmodium falciparum *malaria. Nature.

[B2] Hay SI, Guerra CA, Tatem AJ, Noor AM, Snow RW (2004). The global distribution and population at risk of malaria: past, present, and future. Lancet Infect Dis.

[B3] Breman JG, Alilio MS, Mills A (2004). Conquering the intolerable burden of malaria: what's new, what's needed: a summary. Am J Trop Med Hyg.

[B4] May J, Meyer CG (2003). Chemoresistance in falciparum malaria. Trends Parasitol.

[B5] Plowe CV (2005). Antimalarial drug resistance in Africa:strategies for monitoring and deterrence. Curr Top Microbiol Immunol.

[B6] World Health Organization, Roll Back Malaria Department Current WHO Recommendations on malaria control.

[B7] Alonso PL, Sacarlal J, Aponte JJ, Leach A, Macete E, Milman J, Mandomando I, Spiessens B, Guinovart C, Espasa M, Bassat Q, Aide P, Ofori-Anyinam O, Navia MM, Corachan S, Ceuppens M, Dubois MC, Demoitié MA, Dubovsky F, Menéndez C, Tornieporth N, Ballou WR, Thompson R, Cohen J (2004). Efficacy of the RTS,S/AS02A vaccine against *Plasmodium falciparum *infection and disease in young African children: randomised controlled trial. Lancet.

[B8] Schellenberg D, Menendez C, Kahigwa E, Aponte J, Vidal J, Tanner M, Mshinda H, Alonso P (2001). Intermittent treatment for malaria and anaemia control at time of routine vaccinations in Tanzanian infants: a randomised, placebo-controlled trial. Lancet.

[B9] White NJ (2005). Intermittent presumptive treatment for malaria. PLoS Med.

[B10] O'Meara WP, Breman JG, McKenzie FE (2005). The promise and potential challenges of intermittent preventive treatment for malaria in infants (IPTi). Malar J.

[B11] Chandramohan D, Owusu-Agyei S, Carneiro I, Awine T, Amponsa-Achiano K, Mensah N, Jaffar S, Baiden R, Hodgson A, Binka F, Greenwood B (2005). Cluster randomised trial of intermittent preventive treatment for malaria in infants in area of high, seasonal transmission in Ghana. BMJ.

[B12] Macete E, Aide P, Aponte J, Sanz S, Mandomando I, Espasa M, Sigauque B, Dobano C, Mabunda S, Dgedge M, Alonso P, Menendez C (2006). Intermittent preventive treatment for malaria control administered at times os routine vaccinations in Mozambican Infants: a randomized, placebo-controlled trial. J Infect Dis.

[B13] Kobbe R, Kreuzberg C, Adjei S, Thompson B, Langefeld I, Thompson PA, Abruquah HH, Kreuels B, Ayim M, Busch W, Marks F, Amoah K, Opoku E, Meyer CG, Adjei O, May J (2007). A Controlled Trial on Extended Intermittent Preventive Antimalarial Treatment of Infants. Clin Infect Dis.

[B14] Kreuels B, Kobbe R, Adjei S, Kreuzberg C, von Reden C, Baeter K, Klug S, Busch W, Adjei O, May J (2007). Spatial variation of malariaincidence in young children from a geographically homogeneous areawith high endemicity. J Infect Dis.

[B15] Kobbe R, Neuhoff R, Marks F, Adjei S, Langefeld I, Adjei O, Meyer CG, May J (2006). Seasonal variation and high multiplicity of first *Plasmodium falciparum *infections in children from a holoendemic area in Ghana, West Africa. Trop Med Int Health.

[B16] Marks F, Evans J, Meyer CG, Browne EN, Flessner C, von Kalckreuth V, Eggelte TA, Horstmann RD, May J (2005). High prevalence of markers for sulfadoxine and pyrimethamine resistance of *Plasmodium falciparum *in the absence of drug pressure in the Ashanti region of Ghana. Antimicrob Agents Chemother.

[B17] Schreiber N, Kobbe R, Adjei S, Adjei O, Klinkert M, May J (2007). Immune responses after single dose sulfadoxine-pyrimethamine indicate underestimation of protective efficacy of intermittent preventive treatment in infants. Trop Med Int Health.

[B18] Hosmer DW, Lemeshow S (1999). Applied Survival Analysis.

[B19] Marsh K, Otoo RJ, Hayes RJ, Carson DC, Greenwood BM (1989). Antibodies to blood stage antigens of Plasmodium falciparum in rural Gambians and their relation to protection against infection. Trans R Soc Trop Med Hyg.

[B20] Snow RW, Molyneux CS, Warn PA, Omumbo J, Nevill CG, Gupta S, Marsh K (1996). Infant parasite rates and immunoglobulin M seroprevalence as a measure of exposure to *Plasmodium falciparum *during a randomized controlled trial of insecticide-treated bed nets on the Kenyan coast. Am J Trop Med Hyg.

[B21] Drakeley CJ, Corran PH, Coleman PG, Tongren JE, McDonald SL, Carneiro I, Malima R, Lusingu J, Manjurano A, Nkya WM, Lemnge MM, Cox J, Reyburn H, Riley EM (2005). Estimating medium- and long-term trends in malaria transmission by using serological markers of malaria exposure. Proc Natl Acad Sci USA.

[B22] Francis D, Nsobya SL, Talisuna A, Yeka A, Kamya MR, Machekano R, Dokomajilar C, Rosenthal PJ, Dorsey G (2006). Geographic differences in antimalarial drug efficacy in Uganda are explained by differences in endemicity and not by known molecular markers of drug resistance. J Infect Dis.

[B23] Greenwood B (2007). Intermittent preventive antimalarial treatment in infants. Clin Infect Dis.

[B24] Hay SI, Rogers DJ, Toomer JF, Snow RW (2000). Annual *Plasmodium falciparum *entomological inoculation rates (EIR) across Africa: literature survey, Internet access and review. Trans R Soc Trop Med Hyg.

[B25] Greenwood B (1989). The microepidemiology of malaria and its importance to malaria control. Trans R Soc Trop Hyg.

[B26] Smith DL, Dushoff J, McKenzie FE (2004). The risk of a mosquito-borne infection in a heterogeneous environment. PLoS Biol.

[B27] Smith DL, Dushoff J, Snow RW, Hay SI (2005). The entomological inoculation rate and *Plasmodium falciparum *infection in African children. Nature.

[B28] Filion GJ, Paul RE, Robert V (2006). Transmission and immunity: the importance of heterogeneity in the fight against malaria. Trends Parasitol.

[B29] Greenwood B (2006). Intermittent preventive treatment – a new approach to the prevention of malaria in children in areas with seasonal malaria transmission. Trop Med Int Health.

[B30] McDonald G (1957). The epidemiology and control of malaria.

[B31] Beales FP, Gilles HM, Warrell DA, Gilles AGHM (2002). Rationale and technique of malaria control. Essential Malariology.

[B32] Molineaux L, Wernsdorfer WH, McGregor Sir I (1989). The epidemiology of human malaria as an explanation of its distribution, including some implications for its control. Malaria: Principles and practices of malariology.

